# MR‐guided 125I seed implantation treatment for maxillofacial malignant tumor

**DOI:** 10.1002/acm2.13112

**Published:** 2020-12-09

**Authors:** Ying Wang, Peng Kang, Wei He, Rui Li

**Affiliations:** ^1^ Department of Stomatology The First Affiliated Hospital of Zhengzhou University Zhengzhou China

**Keywords:** ^12I5^, brachytherapy, maxillofacial malignancies, MR‐guided, seed implantation

## Abstract

**Purpose:**

This study assessed the therapeutic efficacy of postoperative magnetic resonance (MR)‐guided interstitial ^125^I seed implantation for treatment of oral and maxillofacial malignant tumors.

**Methods and Materials:**

A total of 127 patients with oral or maxillofacial malignant tumors were included in this study who received interstitial ^125^I treatment after the surgery resection. Before implantation, all the patients received MR scans to assess the lesion scope, extent, and nature. ^125^I implantation target regions were based on the pre‐operative imaging. ^125^I seeds were delivered to target regions via puncture needles under the real‐time guidance of MR. Computed tomography (CT)or MR was performed immediately after implantation and again every 3 months later.

**Results:**

After successful ^125^I implantation, all patients were also examined regularly to detect tumor recurrence, lymphatic, and distant metastases. To date, CT or MR verification showed that 13/127 patients experienced tumor recurrence or lymphatic metastasis or distant metastasis. No seeds migration was observed, no serious treatment‐related complications affected patient quality of life, and no important organ (such as major cervical vessels, spinal cord, etc.) injuries were observed.

**Conclusion:**

Our results show that MR‐guided ^125^I implantation is an effective approach to site‐specific treatment for oral and maxillofacial tumor, which could potentially reduce postoperative complications and tumor recurrence rates, increase patient survival, and improve quality of life.

**Abbreviations**
MR: magnetic resonance
CT: Computed tomography
TPS: treatment planning system
PTC: percutaneous transhepatic cholangiography
MPD: matched peripheral dose

## 1 INTRODUCTION

More than 30% of oral and maxillofacial tumors are malignant.^1^, ^2^ While surgical operations remain the most important therapeutic method, surgery combined with radiotherapy, and/or chemotherapy has gradually become the main treatment for these malignant tumors.^3^, ^4^, ^5^ However, the oral and maxillofacial region is anatomically and histologically complex, and tumor excision frequently injures important structures, such as facial nerves.^6^ Patients’ quality of life may be reduced if these structures lose the physiological function. Although the conventional external beam radiotherapy can reduce tumor recurrence rate, this treatment method also damages normal tissues and organs, resulting in severe complications, such as skin fibrosis, oral mucositis, oral ulcer, hemorrhage, or osteoradionecrosis (ORN).^7^, ^8^, ^9^

^125^I brachytherapy is clinically efficient against malignancies, which can reduce complications from the conventional external beam radiotherapy and improve patient quality of life.^10^, ^11^, ^12^ To achieve optimal clinical therapeutic effects, the ^125^I implantation plan implemented in the present study was designed by a treatment planning system (TPS) with patient imaging data. ^125^I was delivered to a pre‐operatively designated target region under real‐time magnetic resonance imaging (MR) guidance.^13^ Accordingly, the target region received stable, short‐range radiation, with scarce damage to normal tissues.^14^, ^15^ During conventional computed tomography (CT)‐guided surgery, artifacts from the metal puncture needle used for ^125^I implantation can interfere with lesion images. The present study used MR due to its enhanced sensitivity without artifacts compared to CT. The MR‐guided technique was proved to have good therapeutic effects in the treatment of lung and liver cancers.^16^, ^17^ This study retrospectively reviewed patients who underwent complete resection of oral or maxillofacial malignant tumor and following MR‐guided ^125^I brachytherapy in the Department of Oral and Maxillofacial Surgery, the First Affiliated Hospital of Zhengzhou University. Then the clinical therapeutic efficacy of this treatment regimen was assessed to provide references for clinical work.

## 2 MATERIALS AND METHODS

### 2.1 Patients

Between February 2012 and February 2017, 127 patients received surgical resection in the Department of Oral and Maxillofacial Surgery, the First Affiliated Hospital of Zhengzhou University. MR‐guided ^125^I brachytherapy was operated 7 days after surgery. Before ^125^I brachytherapy, no patient had lymphatic or distant metastasis. There were 70 men and 57 women, aged 12–85. The list of histologies is in the Table 1. Inclusion criteria: We evaluated the general condition of patients before operation, including blood pressure, cardiopulmonary function, and whether they could tolerate the general anesthesia. Patients were in good condition with normal liver and kidney functions, and could tolerate radioactive ^125^I brachytherapy. Meanwhile, patients who suffered from benign or recurrent tumors or in poor physical condition were excluded.

**TABLE 1:** Patient demographics.

Measure	n	%
Nationality: Chinese,		
Min. Age: 12,		
Max. Age: 85,		
Sex		
Men	70	55.12
Women	57	44.88
Total	127	100
Number of seeds		
Min: 10,		
Max: 125,		
Mean: 40.79,		
Pathological types		
Squamous‐cell carcinoma	15	11.81
Adenoid cystic carcinoma	29	22.83
Mucoepidermoid carcinoma	31	24.41
Acinar cell carcinoma	6	4.72
Basal cell carcinoma	2	1.58
Malignant polymorphous‐ adenocarcinoma	5	3.94
Epithelial‐myoepithelial‐ carcinoma	8	6.30
Ductal carcinoma	6	4.72
Small cell carcinoma	6	4.72
Oncocytic carcinoma	5	3.94
Chondrosarcoma	5	3.94
Fusocellular sarcoma	3	2.36
Fibrosarcoma	2	1.58
Adenocarcinoma	4	3.15
Total	127	100
TNM staging		
T_1_	42	33.07
T_2_	50	39.37
T_3_	35	27.56
Total	127	100
Results		
Significant effect	114	89.76
Lymphadenectasis	9	7.09
Distant metastasis	3	2.36
Recurrence	1	0.79
Total	127	100

### 2.2 Materials and devices

**FIG. 1:**
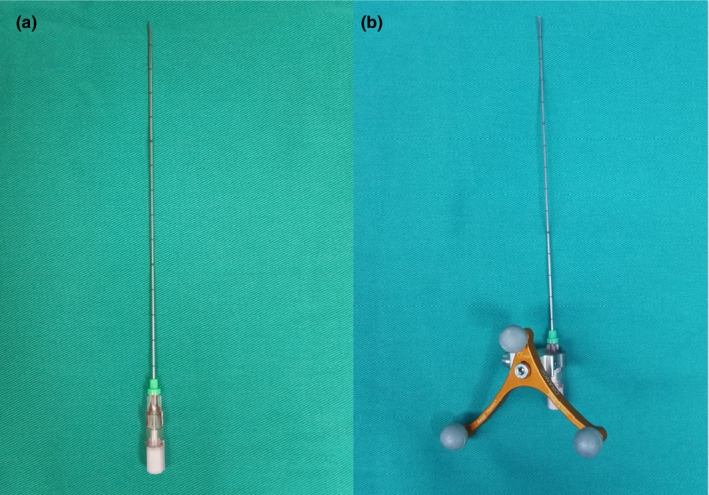
PTC needle (a) used to deliver radioactive ^125^I seeds into the pre‐operatively designated target region. ^125^I radioactive seeds‐loaded PTC needle (with outer shell)(b): PTC needle and its outer shell could become a combination, and the combination was registered in the MR navigation system. We put ^125^I seeds into the metal puncture needle, and the position of the metal puncture needle and ^125^I seeds could be recognized by MR navigation system.

^125^I (produced by Shanghai Xinke Corporation, is cylindrical in shape (0.8 ± 0.05 mm in diameter and 4.5 ± 0.5 mm in length) with titanium alloy casing, average energy 27.4‐35.5 kV, half‐life 59.6d and penetrating distance 1.7 cm. Radioactive ^125^I seed is delivered to the target region via a puncture needle (Hakko Co., Ltd.) [Fig. 1(a)] with an outer shell [Fig. 1(b)] which can be recognized by the navigation system under real‐time MRI guidance (3.0T MAGNETOM Verio MRI; Siemens, Germany).

### 2.3 ^125^I implantation

**FIG. 2:**
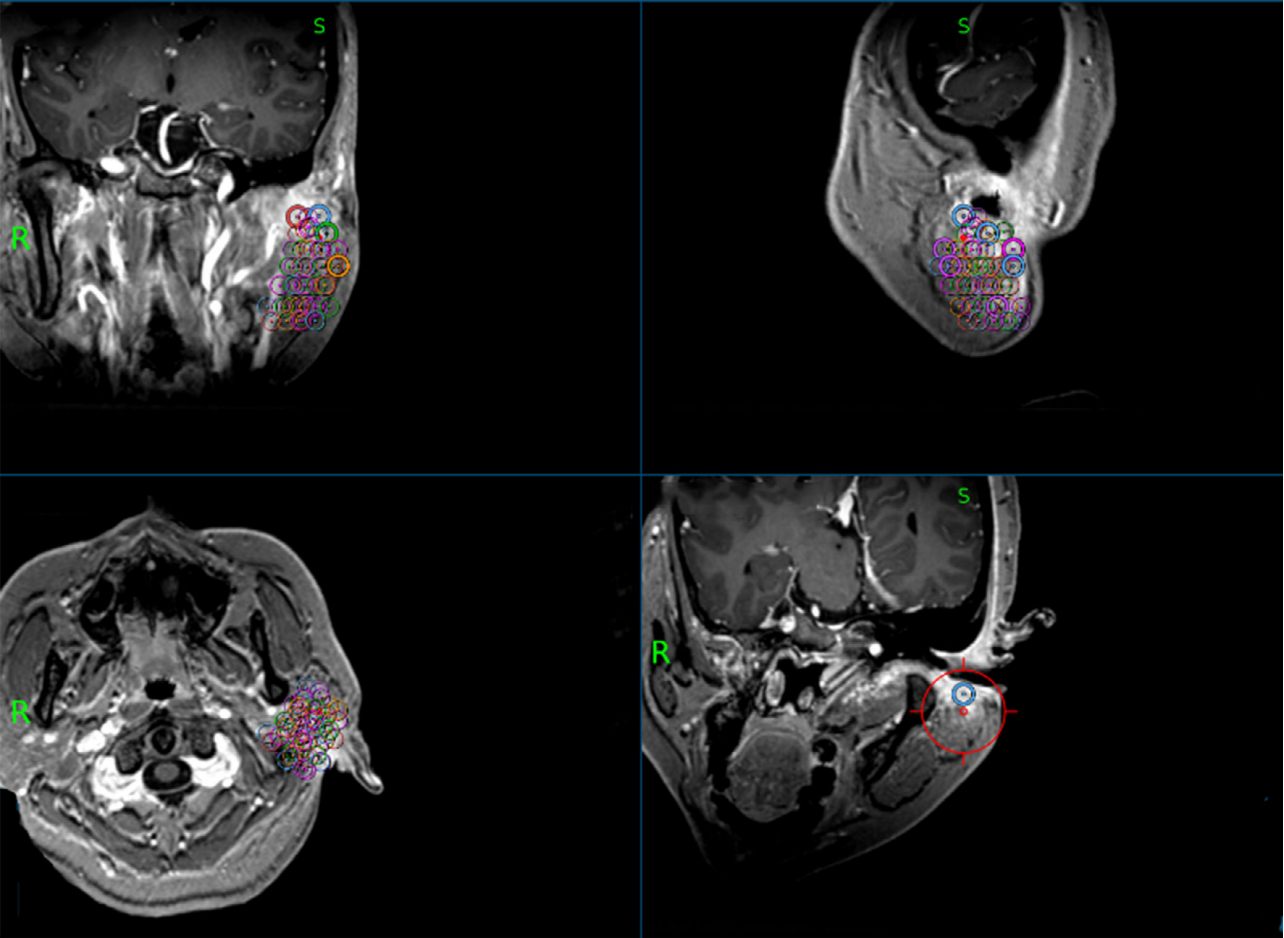
^125^I implantation target regions can be designated based on pre‐operative patient MRIs.

**FIG. 3:**
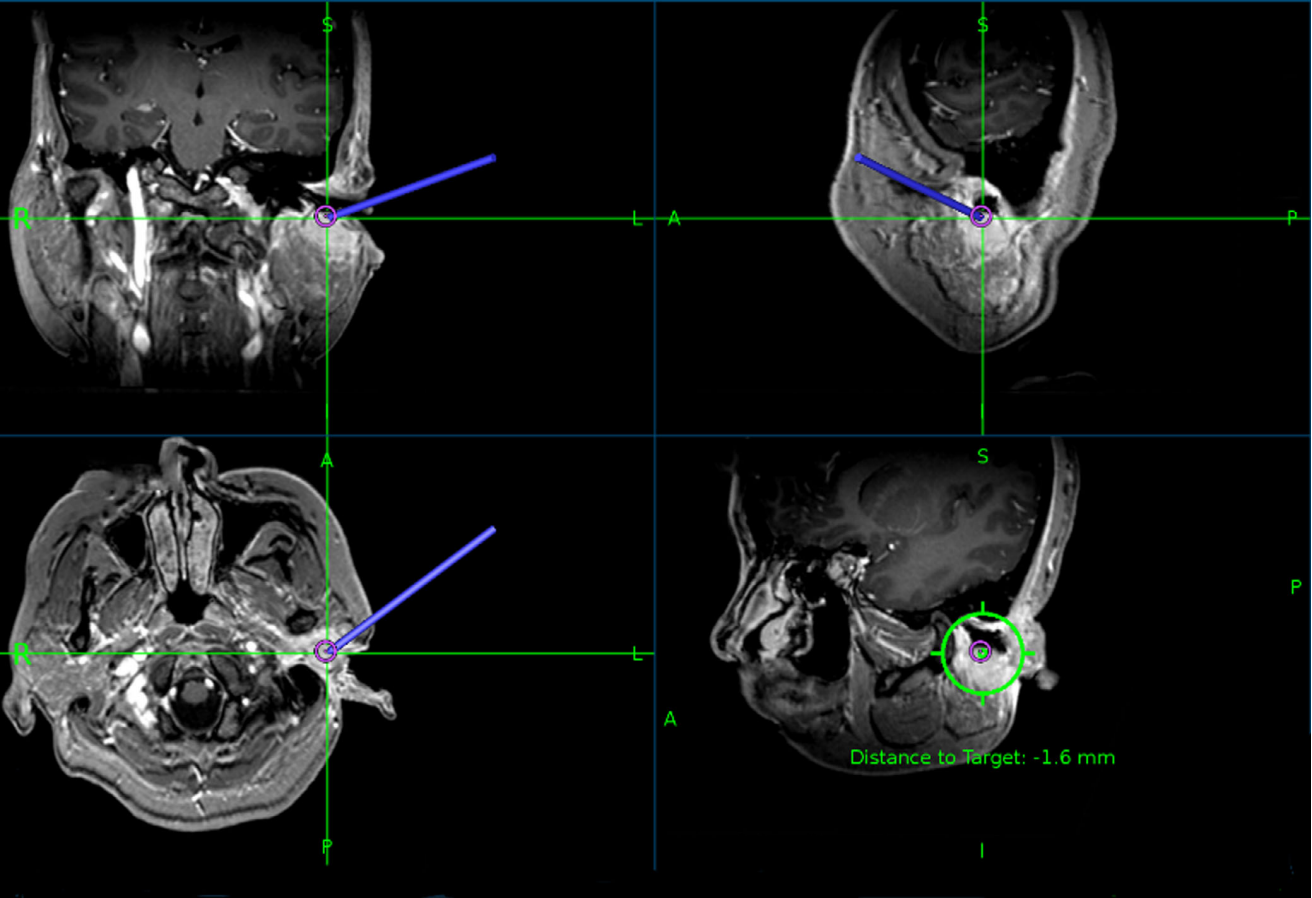
^125^I seeds were implanted in the target region via puncture needle, using real‐time guided MR.

Before surgery, all the patients received MR scans to assess lesion scope, extent, and nature. We designated the target regions according to MR (Fig. 2). Radioactive ^125^I seeds were delivered to these target regions via puncture needles under real‐time MR guidance (Fig. 3). The MR scanning sequence was T1 MPRAGE (TR: 1900 ms; TE:2.93 ms; Flip angle:9; Scan matrix: 2562215; Slice thickness:1 mm). ^125^I seed activity was 0.7 mCi. The matched peripheral dose (MPD) was 60 Gy. The reference point of the implantation was located 0.5 cm outside the target area, and the dose was 90% of the isodose line. Seed implantation was performed according to the Paris principles at the depth of 10 mm, and the space between each implantation was 1 cm. Postoperation CT scans were input into the TPS to detect seed locations and distributions. If seeds were poorly positioned, the radiotherapy efficacy could be reduced, then a group of extra seeds was implanted. The MPD was 60 Gy, D90 was >80 Gy, and V150 was <50%.

### 2.4 Post‐implantation verification and re‐examination

CT scans were performed 48 h after implantation, and the data were added to the TPS. The verification of implanted seed locations and distributions was accomplished by comparing pre‐implantation and post‐implantation data of CT and MR.^18^, ^19^ Once the “cold spots” (the pre‐designed regions with no seeds) were found, the re‐implantation would be conducted in time. Then patients were observed every 3 months to assess the therapy efficacy. If there were no abnormalities during the first postoperative year, patients were assessed every 6 months thereafter. In addition to the routine physical examination, cervical ultrasound was performed to detect the possible lymph node metastasis. For adenoid cystic carcinomas, chest X rays were performed to detect lung metastases. For those patients suspicious for regional recurrence, CT scans were performed and compared with pre‐operative MR.

### 2.5 Quality of life evaluation

The quality of life (QOL) of these patients was assessed by using the European Organization for Research and Treatment of Cancer Quality of Life Questionnaire Head and Neck Module (EORTC‐QLQ‐H&N35)before surgical resection and at 3 and 12 months after operation. The results were statistically analyzed by one‐way ANOVA and paired t‐test.

## 3 RESULTS

The average follow‐up period was 54.6, and 36 months was the minimum for a case to be included in the series. In total, 127 patients received ^125^I implantation in our study (Table 1). The number of seeds varied from 10 to 145 (average, 40.79). No “cold spots” were discovered. Post‐implantation CT verifications showed that all seed distributions and positions were consistent with pre‐operatively designated target areas. There were no cases of seed migration.

**FIG. 4:**
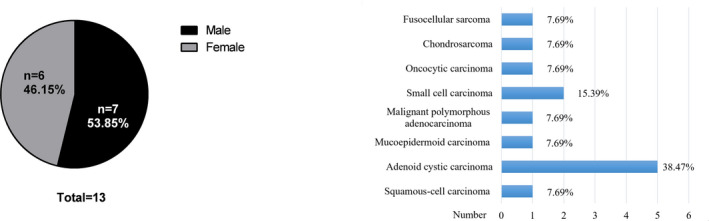
Distribution of recurrence and metastasis data.

**FIG. 5:**
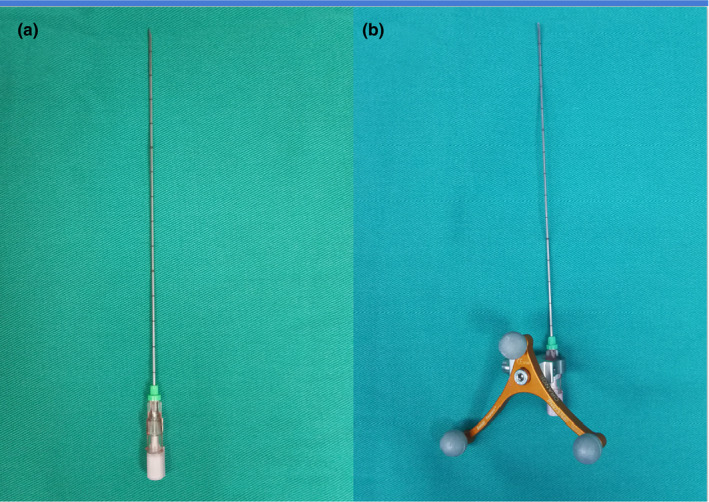
Pre‐(a)and post‐implantation (b)MR comparisons showed that lesions were reduced in size following treatment.

Only 13/127 cases experienced tumor recurrence or metastasis (Fig. 4), with a local control rate of 89.76% (Table 2). Three cases experienced distant metastasis and died (maxilla small cell carcinoma in maxillary bone, male, 85 years old; fusocellular sarcoma in right cheek, female, 44 years old; oral adenoid cystic carcinoma, female, 61 years old). One case experienced recurrence (left tempora small cell carcinoma, female, 12 years old). And 113/127 cases showed lesions were reduced in size following treatment (Fig. 5). There were nine cases of lymphatic metastasis without obvious discomfort.

**TABLE 2:** Recurrence or metastasis patient demographics

Measure	n	%
Sex		
Male	7	53.85
Female	6	46.15
Total	13	100
Pathological types		
Squamous‐cell carcinoma	1	7.69
Adenoid cystic carcinoma	5	38.47
Mucoepidermoid carcinoma	1	7.69
Malignant polymorphous adenocarcinoma	1	7.69
Small cell carcinoma	2	15.39
Oncocytic carcinoma	1	7.69
Chondrosarcoma	1	7.69
Fusocellular sarcoma	1	7.69
Total	13	100
Location		
parotid gland	7	53.85
tempora	1	7.69
maxilla	1	7.69
condyle	1	7.69
buccalmucos	2	15.39
palate	1	7.69
Total	13	100
Results		
Lymphadenectasis	9	69.23
Distant metastasis	3	23.08
Recurrence	1	7.69
Total	13	100

In this study, EORTC QLQ‐H&N35 quality of life assessment scales were made for these 127 patients. One hundred and twenty‐seven questionnaires were sent out and 121 of them were recovered. One hundred and fifteen questionnaires were valid, with an effective rate of 95.04% (115/121). The scores of the QOL before operation, 3 and 12 months after operation were 42.6 ± 6.1, 40.48 ± 8.9, and 30.48 ± 8.1. Compared with the QOL at pre‐operation, swallowing function, language function and dietary status, and social contact of patients were changed insignificantly at 3 months later after operation (*P *> 0.05). But scores of social contact of patients were reduced (*P* < 0.05) compared to pre‐operation. Scores of the ache caused by the focus were reduced at 12 months later after operation, compared with pre‐operation. According to the RTOG/EORTC grading standard for radiation injury, the incidence of acute and terminal radiation damage that significantly decreased patients’ quality of life was zero. No patients had acute inflammation, ulcer, or hemorrhage of skin and mucosa, xerostomia, osteoradionecrosis, seed migration, or damage to important tissues and organs.

## 4 DISCUSSION

Surgical excision is the conventional treatment for oral and maxillofacial malignant tumors.^20^, ^21^ Postoperative radiotherapy is usually applied for high‐grade malignant tumors and nerve‐involved tumors.^22^ Compared with surgical resection alone, surgical excision plus postoperative radiotherapy can greatly increase oral and maxillofacial malignancy local control and long‐term survival rates.^5^, ^23^ However, the conventional external beam radiotherapy can seriously damage normal tissues, leading to oral mucositis, hemorrhage, xerostomia, radiation caries and orradionecrosis of the jaws.^11^, ^24^ Precision radiotherapy aims to improve radiation curative effects and reduce side effects by targeting lesions with more precise radiation doses and protect normal tissues. The ^125^I radionuclide has low photon energy, and a short effective penetration distance in tissues,^25^, ^26^ and ^125^I is now commonly used as a supplementary therapy for malignant tumors, including lung, rectal, liver, breast, and cervical cancers, osteosarcomas, and others.^27^, ^28^, ^29^, ^30^

The curative effects of brachytherapy are mainly determined by the precise assessment of target lesion positioning, precise distribution of the radiation dose and an accurate radiation plan. The most critical aspect of the treatment plan is the precise implantation of radioactive seeds to protect normal tissues. While CT‐guided implantation was used widely in the past,^31^, ^32^, ^33^, ^34^ MR has higher spatial and density resolutions and can clearly show lesion shape, size, and anatomic relationship with the surrounding tissues. MR can also reconstruct three‐dimensional images by basing on cross‐sections, providing more accurate targeting. In addition, the scattering of the metal puncture needle could hindered exact positioning of lesions at CT‐guided radioactive ^125^I implantation.^35^, ^36^ As MR has enhanced sensitivity compared to CT, it can provide real‐time guidance during radioactive ^125^I implantation, and allow puncture needle insertion at a safe and exact angle, depth, and position, and prevent important structures from injury. MR imaging can also be used for verification after implantation. Once “cold spots” are discovered, seeds can be re‐added immediately. Then MR can be used to monitor radioactive seeds distribution and assess treatment efficacy.

Additionally, MR scans do not cause ionizing radiation injuries in patients. The MR vascular flow‐void technique (black and bright technique) and enhancement effect could clearly show oral and maxillofacial blood vessels and nerves and their relationships with lesions, without contrast enhancement. MR navigation systems are also easy to be operated, having fast scanning speeds and short imaging times, and are suitable for radioactive seeds implantation at oral and maxillofacial tumor sites. MR guidance can not only improve puncture and implantation safety and accuracy, but also effectively shorten operation time. We combined pre‐operative MR with the TPS which accurately defined patient tumor extent, location and relationship with surrounding tissues and enabled implantation of MR‐guided radioactive seeds more accurately. ^125^I seeds were delivered to pre‐operatively designated target sites safely and accurately, without major negative impacts to normal tissues. During follow‐up, seed distributions remained consistent with pre‐operative treatment plans, no seed migration was observed, and the local tumor control was satisfactory. MR guidance therefore enables additional therapeutic opportunities for patients. However, MR application is still restricted due to high device and examination expenses, and interference by pulsing blood vessels near lesions. Novel technological developments are needed to overcome these limitations.

Importantly, the factors such as tumor type, patient clinical characteristics, biological features, and pathological results must be considered in order to pinpoint ideal target regions for ^125^I implantation. In our study, 13 patients experienced tumor recurrence and metastasis. This may have been due to poor overall physical condition, the presence of previously undiagnosed lesions, or insufficient radiation dose or effective range. Twelve months after the implantation, EORTC QLQ‐H&N35 quality of life assessment scores showed that social contact and ache caused by the focus of patients were reduced compared to pre‐operation, which suggested that patients could have better social interaction and quality of life after ^125^I brachytherapy.

## 5 CONCLUSION

^125^I brachytherapy for the treatment of oral and maxillofacial malignant tumors can effectively reduce local recurrence rates, increase patient survival, and improve quality of life.^35^, ^36^ MR guidance enables precise radioactive seed delivery to target regions and reduces radiation side effects in normal tissues. Future studies using MR‐guided ^125^I seed implantation should focus on additional tumor types with larger patient cohorts.

## FUNDING

The study was supported by the National Natural Science Foundation of China [31670994], Nature science fund of Henan province [182300410340].

## CONFLICT OF INTEREST

The author have no other relevant conflicts of interest to disclose.
