# Corrigendum: MR‐guided 125I seed implantation treatment for maxillofacial malignant tumor

**DOI:** 10.1002/acm2.13272

**Published:** 2021-05-12

**Authors:** Ying Wang, Peng Kang, Wei He, Rui Li

**Affiliations:** ^1^ Department of Stomatology The First Affiliated Hospital of Zhengzhou University Zhengzhou China

In the article cited above, the published version of Figure 5 was incorrect, as image provided for Figure 1 was printed again under the caption Figure 5. The corrected version of Figure 5 is shown below.

**Fig. 5:**
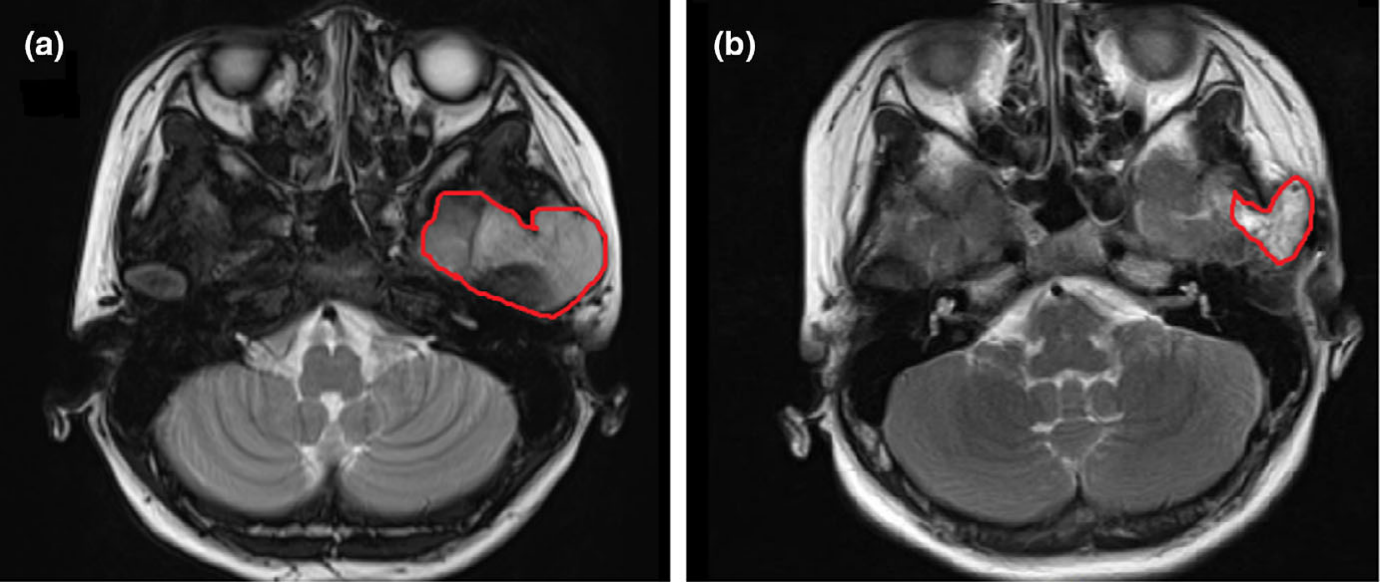
Pre‐ (a) and post‐implantation (b) MR comparisons showed that lesions were reduced in size following treatment.

